# The Impact of Walking on BDNF as a Biomarker of Neuroplasticity: A Systematic Review

**DOI:** 10.3390/brainsci15030254

**Published:** 2025-02-27

**Authors:** Mohamed Hesham Khalil

**Affiliations:** Department of Architecture, University of Cambridge, Cambridge CB2 1PX, UK; mhmhk2@cam.ac.uk

**Keywords:** neurosustainability, brain-derived neurotrophic factor, BDNF, walking, step count, exercise, physical activity, neuronal growth and survival, synaptic plasticity

## Abstract

Background/Objectives: The brain-derived neurotrophic factor (BDNF) is a critical exercise-induced modulator of various neuroplasticity processes, including adult hippocampal neurogenesis. Environmental affordance for physical activity is a novel theory that aims to increase the BDNF through walking or climbing stairs, stimulated by the urban and interior environment. In a systematic review, this paper explores the association between walking, as a structured or free-living form of physical activity, and changes in the BDNF in humans with healthy locomotion. Method: A systematic review with a registered protocol, INPLASY2024110093, and following the PRISMA guidelines, includes English-language original research articles on adult and older adult human subjects who are locomotor-healthy, studies on walking as a structured exercise or free-living physical activity that is presented in a non-combined intervention, and must report changes in the BDNF as a dependent variable. The search was conducted using three databases: PubMed, Web of Science, and Scopus, resulting in 21 eligible studies. Results: This systematic review finds that the impact of walking on the BDNF is evidenced, but subject to moderate to high intensities in single bouts. At the same time, the long-term effects are yet to be fully understood, potentially due to the uptake of the BDNF for functional brain improvements, neuroplasticity processes, or muscle repair, instead of an accumulation of the BDNF itself, yet still confirm the important role of the BDNF for neurosustainability. Age and environmental factors such as heat are also found to affect the increase in the BDNF. The narrative synthesis provides elaborate explanations for understanding those complex dynamics before reaching future conclusions on the impact of walking or environmental affordance for physical activity on the changes in the BDNF concentrations. Conclusions: This systematic review highlights the potential role played by moderate- and high-intensity walking as a lifestyle intervention that can be utilised through the built environment to promote adaptive brain changes, through the sustainable regulation of the BDNF.

## 1. Introduction

Walking is a cost-free and effective habit that has been shown to promote adaptive structural neuroplasticity in the hippocampus [1]. However, because structural changes are long term, the hypothesis that the physical environment can consistently support adaptive neuroplasticity and neurosustainability [2] is promising. Neurosustainability refers to the sustained promotion of adaptive neuroplasticity and growth factors, primarily driven by physical activity [3,4], but structural plasticity does not occur instantaneously. The brain-derived neurotrophic factor (BDNF) plays a central role in linking growth factors and neurotransmitters to adaptive neuroplasticity [4]. Notably, the BDNF responds rapidly to brief physical activity sessions lasting less than an hour (e.g., 20–35 min) [5,6], unlike its slower response to other environmental enrichment factors such as cognitive training and mindfulness [4]. This paper addresses a critical gap in the literature by examining the relationship between environmental affordances for physical activity [3] and the impact of walking on adaptive hippocampal structural plasticity [1]. Through a systematic review, it investigates how single bouts of short-term and long-term walking influence the BDNF concentrations in healthy adults and older adults with intact locomotion.

Research shows that the BDNF responds to individual exercise sessions, and that this response becomes stronger with consistent exercise participation [7,8,9]. Higher exercise intensity leads to greater BDNF production, though this relationship depends on one’s fitness level [10]. While the optimal exercise type and amount for BDNF production remained uncertain for some time [11], recent evidence indicates that high-intensity interval training is most effective at elevating BDNF levels in adults, with possibly sustained effects [12]. This finding largely aligns with Zhou et al.’s [13] previous analysis, though their review suggested that intensity training surpasses high-intensity interval training in effectiveness.

**Figure 1:**
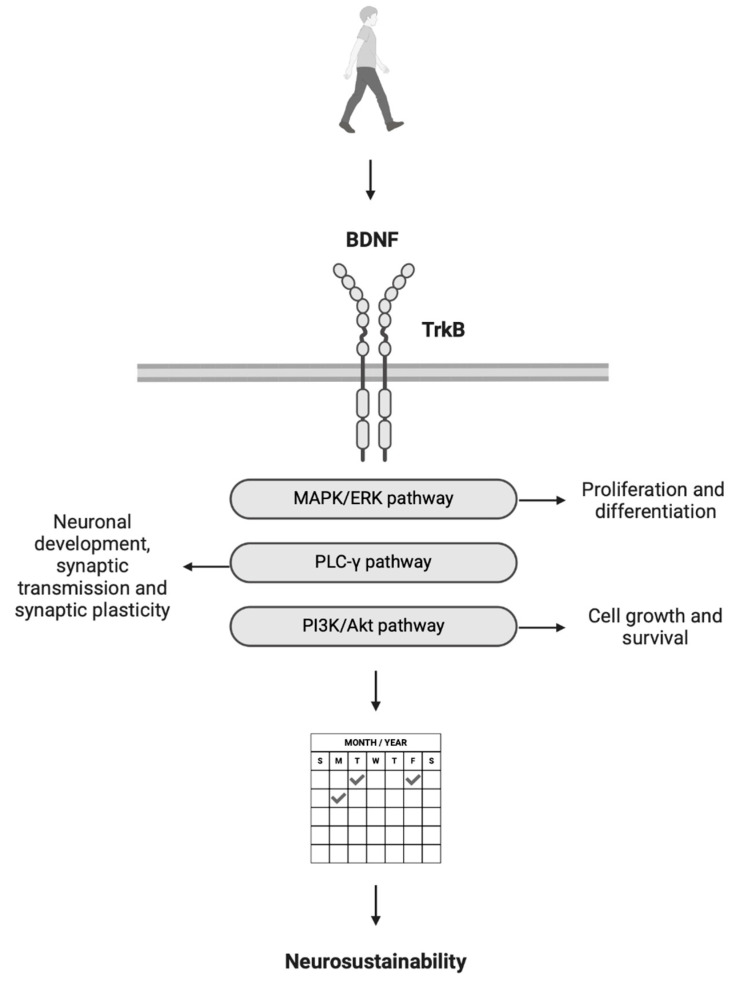
Walking, BDNF and neurosustainability. BDNF modulates neuroplasticity through different signalling pathways for neuroplasticity, allowing a single bout of walking to facilitate neuroplasticity and repeated walking to achieve neurosustainability, which is the sustainable regulation of neuroplasticity through physical activity.

Recent research has explored the BDNF’s critical functions in neurogenesis, neuronal activity, and its relationship to various brain disorders. The BDNF plays a vital role in central nervous system development, including cell differentiation, maturation, and synaptic function [14]. The BDNF gene, which encodes this protein, exhibits complex structural and regulatory characteristics [15,16]. As a key nervous system protein supporting neuronal survival, growth, and synaptic plasticity, the BDNF can be produced in various forms through alternative gene reading. This versatility allows the BDNF to support diverse brain functions, adapting its production based on specific needs during development, learning, or recovery from injury. The process of the BDNF’s action on plasticity involves its initial synthesis as proBDNF, which can then be converted to mature BDNF (mBDNF) [17,18]. Mature BDNF specifically promotes neuronal survival and strengthens synaptic connections, thereby facilitating learning and memory processes. This conversion from proBDNF to mBDNF occurs relatively quickly through physical activity. The BDNF primarily functions by forming a bond with the tropomyosin receptor kinase B (TrkB) receptor [19]. This interaction initiates several internal signalling cascades that support neuronal development, survival, and synaptic plasticity [20,21]. These cascades include the MAPK/ERK pathway, which regulates cell growth, differentiation, and movement [22]; the PI3K/Akt pathway, essential for cell growth, survival, and metabolic functions [23]; and the PLCγ pathway, which influences synaptic plasticity and neural communication [24]. These mechanisms are essential for sustaining adult hippocampal neurogenesis, supporting new neurons from their initial formation to their integration into neural networks. The mediating role of the exercise-induced increase in the BDNF for neurosustainability, which is the sustainability of adaptive neuroplasticity and growth factors through environmental enrichment [3], is illustrated in Figure 1. Walking can become a habitual environment-based physical activity that sustains both the BDNF and potentially adaptive neuroplasticity, as it is already proven to promote adaptive hippocampal formation volume changes [1].

## 2. Method

### 2.1. Research Strategy

Three databases (PubMed, Web of Science, and Scopus) were used for the search in this systematic review. The PRISMA (Preferred Reporting Items for Systematic Reviews and Meta-Analyses) statement and checklist is used for the reporting of all data [25]. The search included all articles found and published up to November 2024, using the following keywords: (BDNF OR “brain-derived neurotrophic factor”) AND (walk* OR “free-living physical activity” OR “step count”). A third search box excluded rodent subject-related keywords: AND NOT (mice OR rat OR rodent OR animal).

### 2.2. Research Framework

The Population, Intervention, Comparison, Outcome (PICO) framework is used to define the inclusion and exclusion criteria at an early stage, before starting the search across the databases [26,27]. Firstly, for Population (P), this systematic review includes adult or older adult human subjects with healthy locomotor ability to observe the changes in the dependent variable without the potential effects of gait or other health-related factors affecting muscles or nerves through walking. Secondly, for the Intervention (I), this systematic review focuses on walking as either a structured exercise or a free-living or lifestyle-based physical activity. Thirdly, regarding the Comparison (C), no comparison was mandatorily required, but in case comparison takes place in any study, the article can be included if it shows the impact of walking independently without combination with other exercise types. It was important to assess the impact of walking without the confounding effects of walking combined with other forms of exercise that could lead to positive effects that may not be attributed to walking itself. Lastly, regarding the Outcome (O), this systematic review is solely interested in the changes in the BDNF concentrations.

### 2.3. Screening and Inclusion Criteria

In addition to the criteria specified through the PICO framework, only English peer-reviewed journal articles were eligible for inclusion, while grey literature references (books, book chapters, conference papers, notes, retracted papers, and reviews) were excluded. A limitation of this systematic review was the inability to search for articles in other foreign languages. Afterwards, duplicates were removed before proceeding with data extraction and the selection of articles for full-text reading based on their title, abstract, and keywords. Any article that was eligible for a full-text read was looked through for more potentially eligible articles. The process was carried out independently.

### 2.4. Risk of Bias Assessment

The Physiotherapy Evidence Database (PEDro) scale was used to assess the risk of bias in randomised controlled trials [28], and since some eligible articles were non-randomised, the risk of bias in non-randomised studies of interventions (ROBINS-I) tool [29] was also used for a fair assessment of all the included studies.

### 2.5. Data Analysis and Synthesis

The collected data from the included studies were analysed and synthesised narratively in order to bridge the current gaps in the literature and provide further useful explanations for future research aiming to test the impact of the physical environment on the BDNF through a single bout of walking, and for future research aiming to explore the associations between walking, the BDNF, and structural plasticity changes.

## 3. Results

**Figure 2:**
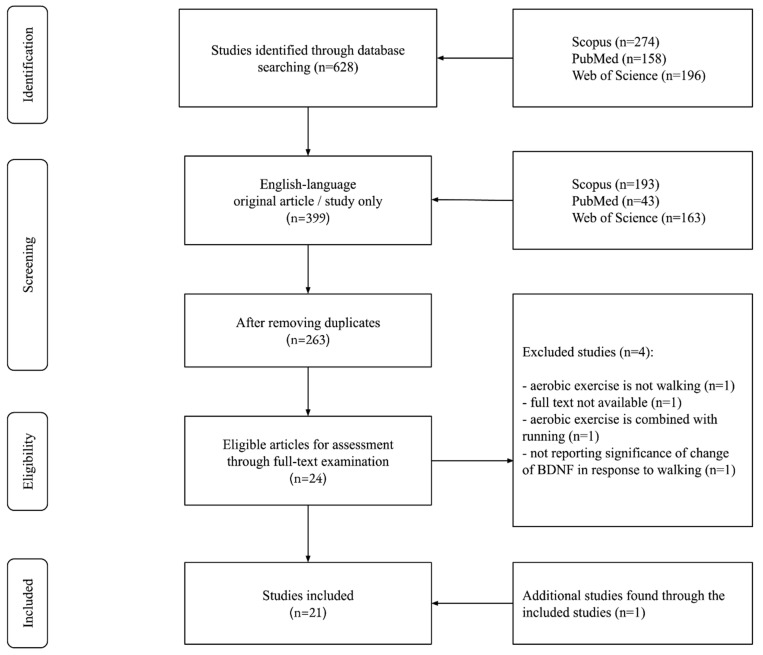
Flow diagram of the study selection process.

Across the 3 databases (Scopus, PubMed, and Web of Science), 628 articles were found, resulting in 263 articles after removing duplicates and considering only original English-language articles. A total of 24 articles were eligible for full-text reading, where 3 articles were excluded for various reasons after meeting the initial inclusion criteria [30,31,32] and 1 article was excluded for not reporting the significance of the increase in walking itself, but respective to vocabulary encoding [33], which is not in line with the aim of this systematic review. By looking through the reference lists and citations of the included studies, 1 article (n = 1) was obtained through one of the included articles [34], resulting in 21 articles at the final stage. The PRISMA flow chart is illustrated in Figure 2. Table 1, Table 2, Table 3 and Table 4 present a summary for the single-bout, short-term (2 weeks), long-term (8–12 weeks), and long-term (24–52 weeks) studies, respectively. The clustering of studies in that regard came out naturally based on the duration gaps identified between the included studies, which turned the limitation into a facilitation of both the micro and macro analysis of the association between walking and the BDNF.

Table 1, Table 2, Table 3 and Table 4 have revealed interesting patterns of not only BDNF changes in response to walking, but also the uptake of the BDNF for functional improvements. Before moving forward onto the discussion of this pattern of BDNF changes, it is important to compare the demographics, significance, effect estimate, and BDNF medium before coming to conclusions. Table 5, Table 6, Table 7 and Table 8 compare the p-values and effect estimates of all included studies, respectively, to Table 1, Table 2, Table 3 and Table 4. Table 9 presents the risk of bias analysis using the ROBINS-I tool for non-randomised trials, while Table 10 uses the PEDro scale. The risk of bias analysis shows acceptable levels based on the given total scores.

## 4. Discussion

This systematic review highlights significant inconsistencies in BDNF responses to walking, including variations in the BDNF levels, its role in adaptive functional neuroplasticity, and its contribution to functional brain improvements. Additionally, BDNF responsiveness appears to be influenced by baseline neurological conditions, environmental conditions, and other factors, further complicating its interpretation. Despite these inconsistencies, the findings reinforce the potential of the BDNF as a biomarker for assessing whether the physical environment can enhance its production. This paper emphasises the need to investigate BDNF responsiveness at the level of a single bout of physical activity. However, for short- and long-term interventions, research should move beyond the BDNF alone and consider adaptive neuroplasticity and functional outcomes as primary dependent variables. Since the BDNF is actively taken up by the brain and plays a metabolic role in supporting other functions, its long-term measurement can be reflected in neuroplasticity and brain health, and not the BDNF itself.

**Figure 3:**
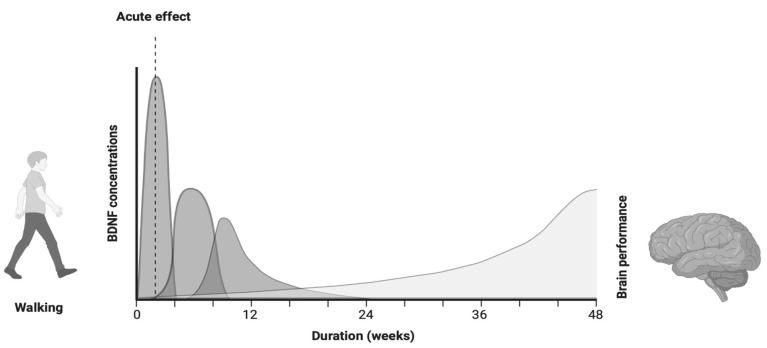
Conceptual relationships between walking duration, BDNF changes, and improved brain performance.

Figure 3 illustrates the complex relationship between the BDNF and improved cognitive function, as suggested through this schematic. However, Figure 3 provides a conceptual illustration of the relationship between walking, the BDNF, and brain health (e.g., improved cognitive function, etc.), while a meta-regression analysis in the future can elucidate those dynamics.

### 4.1. Single Bout Walking and BDNF

There is a modest number of single-bout walking interventions (n = 7), facilitating a narrative synthesis [34,35,36,37,38,39,40].

All single-bout studies used serum BDNF except Silveira-Rodrigues et al. [37], who used plasma BDNF. The duration of the seven studies ranged from 30 min to 8 h, but they were highly variable on subject characteristics. Hutchinson et al. [35] show in their study that a 30 min walk significantly increased the BDNF for both pregnant and non-pregnant women, but we should highlight that gender may have influenced the results. No effect was found on the BDNF when wearing a mask or walking without a mask in another 30 min study [36]. Both studies used serum BDNF, while a 40 min plasma BDNF study showed no significant effect on the BDNF, which could be due to using plasma rather than serum BDNF. Walking for 3.5 h was only significant for the 6 km Nordic walking type and after 40 h [39]. A regular 6 km walk or 18-hole golf round for 3.5 h was not significant enough to increase the BDNF [39], but an 18-hole golf round walk was significant at 5 h in another study [34]. Wheeler et al. [40] showed positive effects on the BDNF increase, but the subjects were overweight, making the parameters given non-generalizable, since it was shown earlier in a systematic review by Khalil [1] that step count promotes adaptive hippocampal plasticity, but subject to health factors such as obesity and type 2 diabetes.

One study showed that walking for 3 h in a hot environment (32 °C) increased the BDNF significantly, but not in a temperate environment [38]. It is worth noting here that the impact of temperature can seriously affect the changes in the BDNF, since Khalil [2] showed in their article on neurosustainability that seasonal variation positively correlates with serum BDNF concentrations [55], that BDNF levels increase by 66% following a 2 min immersion of healthy men in 42 °C water compared to another group immersed in 35 °C water [56], and that rats exposed daily to a 1 h 36 °C heat treatment for 7 days had better hippocampal neurogenesis [57].

Two studies suggested that future experimental models should be of high intensity to promote higher BDNF concentrations [36,39], which explains the significant increase in the BDNF in 5 h but not 3.5 h for the 18-hole golf round, the 6 km Nordic walk but not the 6 km walk, and the significant effect of walking for 3 h in a hot environment (32 °C) but not in a temperate environment.

Still, one study on women still shows that 30 min is enough to significantly elevate the BDNF [35]. It is very important to note that this result may be gender sensitive. The literature shows that a walk in nature for 1 h was significant only among women [58], and that since stress has effects on the BDNF in several brain regions, including the amygdala [59], the effectiveness of 30 min of walking may not be generalizable until exploring it among men and both genders in environments of different temperatures.

Collectively, the seven studies suggest that a single bout of walking either for 30 min, 3.5 to 5 h continuously, or 8 h intermittently can result in variations in the BDNF, while taking into account confounding variables such as temperature, walking intensity, and gender-based amygdala reactivity. With those variables taken into account, future research should use those findings on the impact of a single bout of walking on the BDNF to explore and contrast both walking and high-intensity physical activity (such as using stairs) to further explain the nuanced differences between walking and other habitual physical activities such as cycling or climbing stairs on the BDNF [3].

### 4.2. Short-Term Walking and the BDNF

When moving from the level of a single bout, the effect of the BDNF may not be straightforwardly traceable at the level of the BDNF itself. Short-term studies that lasted for 2 weeks (n = 2) show that walking at moderate intensity, but not at low intensity, was associated with a smaller percentage of WMH volume, but the BDNF levels were not significantly correlated with the WMH volume [41]. The tricky relationship here is that we previously showed that non-low-intensity walking is associated with larger hippocampal volumes [1], and it is evident here that it is associated with WMH volume, but Otsuka et al. [41] explain that the subjects were patients with depressive symptoms and mild cognitive impairment, which affected the expression of the BDNF and may have reduced their association with WMH. Therefore, the relationship between the increased BDNF and neuroplasticity is still not null. Not only consistency for 2 weeks, but also intensity (through inclination) was found to be effective in increasing the BDNF on the last day, not the first [42], which explains that earlier single-bout studies that were insignificant could have had significance if repeated consistently for in the short term. Therefore, at this 2-week intervention duration, researchers can still expect to detect changes in both the BDNF and structural plasticity at the end of the intervention.

### 4.3. Long-Term Walking and the BDNF

Long-term walking interventions (n = 8), 8 to 12 weeks, should be cautiously interpreted. As argued by the researchers of one of the included studies, BDNF reductions can be observed, and that is in the uptake by the brain and its metabolic role for improving cognitive and physical functions [44]. While that conclusion was from a 12-week study, Domaszewska et al. [43] showed that in their 8 weeks of walking intervention, there was a significant decrease in the BDNF in the Nordic walking group, and an increase in the Nordic walking group with a resistance shock absorber, which may suggest that higher intensities can still increase the BDNF while there is a brain uptake. This is because Rodziewicz-Fils et al. [44] showed a reduction in the BDNF for the Nordic walking with resistance poles, but for 12 weeks, not 8, which supports the same hypothesis that there is a threshold for a BDNF increase before it starts to be processed into the brain uptake. Through their 12-week study, Walentukiewicz et al. [49] suggest that changes in the BDNF may signify a reduced BDNF release by the brain or its higher uptake, which suggests that it is very difficult to expect a straightforward BDNF-to-brain relationship. Still, several 12-week studies showed inconsistent results. Gmiąt et al. [50] showed that regular Nordic walking resulted in improvements in cognitive functions and was accompanied by an increase in the BDNF. Rezola-Pardo et al. [47] showed no changes in the BDNF, which was not associated with physical, cognitive, or dual-task parameters. Reed et al. [46] showed no significant main effect on the BDNF, but walking was beneficial in improving physical and mental health for patients. Noushad et al. [45] showed that the BDNF levels decreased after sitting or walking in nature, but there was a significant effect of nature-based walking on traumatic stress and post-traumatic growth. Finally, Caserta et al. [48] showed an increase in the Nerve Growth Factor, but not the BDNF. At this level, through the available evidence, we urge long-term studies to focus on the expected neuroplasticity or functional outcomes rather than the BDNF itself, which may not be representative.

Among the four remaining long-term studies, (n = 1) lasted for 24 weeks, (n = 2) for 1 year, and (n = 1) for 13 months. Chou et al. [51] showed the significant effects on the BDNF, but improvements in total recall, delayed recall, and subjective cognitive impairment, which we see similarly to the 12-week-long studies. Leckie et al. [52] showed that the BDNF mediated the effect of a 1-year long exercise on task-switch performance, but only for subjects older than 71, while Voss et al. [53] showed that the increased temporal lobe connectivity between the bilateral parahippocampal and the bilateral middle temporal gyrus was associated with an increased BDNF and other growth factors through walking. Lastly, Bergman et al. [54] showed that 13 months of light-intensity walking increased the hippocampal volume, but was not mediated by the BDNF, which we can explain as potentially being due to the light-intensity walking activity, which is proven to be ineffective, or that it has been effective, but the BDNF has regulated the adaptive hippocampal plasticity in a non-straightforwardly evident way, as hypothesised earlier.

### 4.4. Gaps and Future Research on the BDNF and Neuroplasticity

The relationship between physical activity (e.g., walking), the BDNF, and neuroplasticity is very complicated. It is still unclear whether long-term walking promotes adaptive neuroplasticity separately, partially or completely via the BDNF, which limits a recommendation on the needed long-term walking programme. The BDNF may not be a long-term dependent variable to look for, but it can still be used as an indicator for the effectiveness of the physical environment to elevate the BDNF after a single bout, but the BDNF itself may not be a direct indicator of those changes, due to the high complexity of the neurophysiological processes.

While this systematic review has highlighted the indirect association between the BDNF increases and long-term positive outcomes, more evidence exists that supports how physical activity has a long-term impact on neuroplasticity. For instance, another recent systematic review on walking shows that walking indeed promotes adaptive hippocampal formation volume changes [1], but that the relationship between walking, the BDNF, and neuroplasticity is still unclear, despite the fact that walking is effective for both. For instance, Bergman et al. [54] showed that walking increased the hippocampal volume, but that the change was not mediated by the BDNF. More research is needed to explain this consistent weak association, despite the fact that the BDNF is known for its role in promoting neuronal cell growth, survival, and synaptic plasticity.

While most of the included studies longer than a single bout reported that walking resulted in positive functional outcomes (e.g., motor, cognitive, etc.), Voss et al. [53] showed in their study that an elevated BDNF resulted in better connectivity between the bilateral parahippocampal and bilateral middle temporal gyrus, which is considered functional, as opposed to structural neuroplasticity.

Nonetheless, more systematic reviews cover the impact of structured exercise on long-term adaptive neuroplasticity, which is related to, but not exclusive to, several neurological diseases. Ploughman et al. [60], through their systematic review of clinical trials and studies in animals, showed that forced exercise at moderate to high intensity elevates the BDNF and other growth factors, such as the nerve growth factor (NGF) and insulin-like growth factor (IGF-1), along with synaptogenesis in different brain regions. More recently, de Sousa Fernandes et al. [61], through their systematic review on both human and animal studies, showed that physical exercise is effective for increasing the production of neurotrophic factors (BDNF, NGF, and GDNF), cell growth, and proliferation, in addition to enhancing brain functionality, which supports the initial hypothesis of this systematic review, and confirms that walking can be considered a form of habitual exercise. Additionally, physical activity and neurotrophic factors were systematically reviewed using meta-analysis, showing how they act as potential drivers for neuroplasticity in Parkinson’s disease [62,63], multiple sclerosis [64,65], Alzheimer’s disease [66,67,68], and neurological pathology [69]. A future systematic review of systematic reviews with meta-analysis would be highly beneficial for exploring the nuanced differences between the impact of walking and other forms of exercise on BDNF concentrations in people with neurodegenerative diseases compared to healthy subjects. Still, these systematic reviews further strengthen the recent hypothesis that physical environment has the potential to be an active promoter of physical activity to boost the BDNF and promote adaptive neuroplasticity [3]. An earlier review suggested that acute exercise bouts would result in high BDNF synthesis which, in turn, can be absorbed more effectively by central or peripheral tissues [70], which is further explained by Khalil [4] in the BDNF-interactive model for sustainable hippocampal neuroplasticity and neurogenesis, where it is argued that BDNF is transported across the blood–brain barrier through saturable and non-saturable transport mechanisms [71,72].

In that regard, environmental affordance for physical activity [3] can help protect against the cognitive decline caused by ageing. Higher amounts, durations, and frequencies of daily walking were found to each be associated with larger volumes of the hippocampus between 0.2 and 1.4%, compared to annual atrophy rates between 0.8 and 2.0% in healthy elders [73]. Subjects with type 2 diabetes, however, may require an additional 15,000 steps to return to a normal hippocampal volume [74]. Future research should be cautious, however, regarding the fact that ageing-related hippocampal volume is associated with a number of neurotoxicity factors besides diabetes, such as clinical depression, bipolar disorder, obesity, hypertension, brain injury, and head traumas [75].

Furthermore, the relationship between physical activity and either the BDNF or neuroplasticity should take into consideration the mediating role of stress and other mental health conditions, which can hinder adaptive neuroplasticity by inhibiting the BDNF regulation [4], or requiring extra physical activity efforts to reach homeostasis. On the one hand, stress affects the BDNF in the hippocampus, amygdala, and cortex [59]. Thus, physical activity and stress have magnitudes of opposite forces, and the sum is going to affect the final BDNF outcome. More physical activity can mitigate the negative effects of stress on the BDNF levels, but chronic exposure to stress can make it difficult for physical activity to be effective without multiplied efforts. On the other hand, the maladaptive neuroplasticity in depression is suggested to be associated with alterations in the BDNF [76,77], which suggests that physical activity can help in overcoming depression. This was shown in a recent pilot study to be more common inside built environments and more specifically in single-storey houses that may lack greater physical activity through the lack of stairs [78]. Hence, physical activity can mitigate both stress and depression, but both can also lead to the design of environments that demand high levels of physical activity. In addition, the increase of BDNF through walking can arguably be an antidote to the complexity of the neurophysiological imbalances in mental health cases such as borderline personality disorder [79].

**Figure 4:**
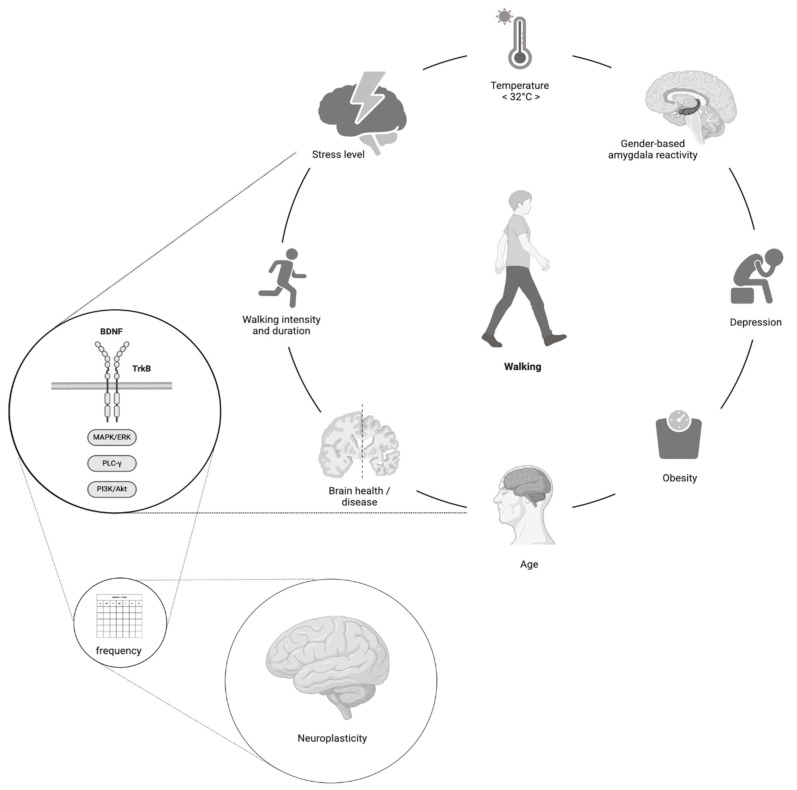
A model for future research on walking, BDNF and neuroplasticity. The relationship between walking and BDNF should be studied separately from or cautiously in conjunction with the relationship between BDNF responses to walking and neuroplasticity changes. Still, the factors affecting the BDNF response to walking are constant.

As illustrated in Figure 4, future research is urged to separately test the impact of walking on the BDNF in a single bout to explore the effect of the environment on elevating the BDNF and the relationship between the BDNF, functional brain improvements, and structural neuroplasticity under the given conditions that affect BDNF concentrations.

## 5. Conclusions

Based on the systematic review of the impact of walking on the brain-derived neurotrophic factor (BDNF) as a biomarker of neuroplasticity, several important conclusions can be drawn. Walking, particularly at moderate to high intensities, has been shown to influence the BDNF levels, with the most acute effects observed shortly after physical activity. However, the long-term effects of walking on the BDNF are less clear, and the brain’s uptake of the BDNF for various neuroplasticity and metabolic processes may limit its accumulation over time, urging the long-term focus to be on adaptive neuroplasticity and functional outcomes instead.

Single-bout interventions can use the immediate responsiveness of the BDNF to test the ability of the environment to induce moderate- and high-intensity walking or other forms of physical activity.

While walking, especially in certain environmental conditions such as heat, can effectively elevate the BDNF, the collective outcomes suggest that a more standardised approach in future research is required. In particular, long-term interventions should focus on broader neuroplasticity and functional outcomes, as the BDNF itself may not be a direct indicator of sustained neuroplasticity.

Further studies should investigate the combined effects of walking and other forms of physical activity, explore varying intensities, and examine the environmental affordances of moderate-to-high intensity walking and possible stair use in promoting BDNF regulation. This review underscores the potential of walking as a sustainable intervention for adaptive neuroplasticity, supporting its integration into daily routines as part of public health and wellbeing strategies.

## Figures and Tables

**Table 1:** Overview of included studies (single bout).

Author/s and Publication Year	Sample (Age)	Study Design (Single Bout)	Results	Discussion Highlights
Hutchinson et al. [35]	Pregnant (n = 13), and non-pregnant (n = 17) women (18 to 40 years old).	30 min of moderate intensity walking (40–60% heart rate reserve).	The BDNF increased in both groups, but was more pronounced in non-pregnancy.	—
Michalik et al. [36]	40 participants (27.6 ± 6.1 years old) who had COVID-19 in the past 6 months.	30 min treadmill walk at 6 km/h (a) wearing a face mask, (b) without a mask.	No statistically significant difference in the BDNF was found.	Future studies need to focus on higher-intensity exercise that can result in higher concentrations of the BDNF.
Silveira-Rodrigues et al. [37]	11 participants (63 ± 7 years old) with type 2 diabetes.	40 min treadmill walk at 4.9 ± 0.9 km/h (90–95% of the max. walking speed).	The walking exercise increased the BDNF by 11%, while the resistance training reduced it by 15%.	Compared to non-diabetic individuals, type 2 diabetes may have a disruptive regulation of the BDNF concentrations.
Goulet et al. [38]	13 healthy active men (22 ± 3 years old), 12 healthy older men (59 ± 4), 10 older men w/hypertension (60 ± 4), and 9 older men with type 2 diabetes (60 ± 5).	180 min treadmill walk of moderate intensity (200 W/m^2^) in temperate (16 °C) and hot environments (32 °C).	No changes in serum BDNF in temperate environments (16 °C), irrespective of group, but serum BDNF was elevated following exercise in the hot environment (32 °C), remaining elevated during recovery (60 min post exercise).	The BDNF response was similar in healthy subjects irrespective of age (up to 63 years old), and was similar among healthy, hypertension, and type 2 diabetes subjects.
Kettinen et al. [39]	25 healthy older adults (69 ± 4 years old).	3.5 h walk (18-hole golf round, 6 km Nordic walking, 6 km walking). Each subject completed one of the three exercises a day with a 1-day washout.	No immediate post-exercise significant changes in the BDNF levels were found between groups or within groups, only in the Nordic walking group after 40 h.	The BDNF’s response to exercise appears to be intensity-dependent, but this study is largely moderate (60–76% HRmax). Reduction in the BDNF can be due to its utilisation for the repair of exercise-induced muscle damage or by the brain during recovery.
Yasuoka et al. [34]	9 recreationally active males (31 ± 4 years old).	18-hole round of golf walk (5807 m) that was completed in 303 ± 4 min.	Serum BDNF concentrations were significantly elevated 1.2 ± 0.3-fold immediately post exercise.	—
Wheeler et al. [40]	67 sedentary overweight/obese older adults (67 ± 7 years old).	8 h of (a) SIT: uninterrupted sitting, (b) EX + SIT: sitting (1 h), moderate intensity walk (30 min), uninterrupted sitting (6.5 h), or (c) EX + BR: sitting (1 h), moderate intensity walk (30 min), interrupted sitting every 30 min with 3 min of light intensity walking (6.5 h).	Serum BDNF was significantly increased in both EX + SIT and EX + BR relative to SIT.	Improvement in serum BDNF and working memory, but the former is not significantly associated with the cognitive outcomes.

**Table 2:** Overview of included studies (short-term of 2 weeks).

Author/s and Publication Year	Sample	Study Design (2 Weeks)	Results	Discussion Highlights
Otsuka et al. [41]	57 participants (>65 years old).	Average daily physical activity and step count were quantified using an accelerometer for 2 weeks.	While not significantly correlated with the BDNF levels, walking speed and higher intensity is associated with white matter hyperintensity (WMH) volume.	Patients had depressive symptoms and mild cognitive impairment, which may have adversely affected the BDNF and also reduced the association between its levels and WMH volume.
Yulinda et al. [42]	20 men in the inclination (31.3 ± 3.04 years old) and speed (32.3 ± 2.31) groups.	2-week experiment (walking inclination and speed groups).	No significant changes in serum BDNF in the inclination group, but a significant increase in the speed group.	—

**Table 3:** Overview of included long-term studies between 8 and 12 weeks.

Author/s and Publication Year	Sample	Study Design (8 to 12 Weeks)	Results	Discussion Highlights
Domaszewska et al. [43]	50 women (60–75 years old).	8 weeks (2 times/week walking 3.5 to 4.5 km), (a) Nordic walking with classic poles or (b) Nordic walking with poles with a resistance shock absorber.	A significant decrease in the BDNF in the Nordic walking group and a non-significant increase in the BDNF in the Nordic walking with resistance shock absorber group.	—
Rodziewicz-Flis et al. [44]	11 (68.7 ± 6 years old).	12 weeks (3 times/week) of Nordic Walking training with BungyPump resistance poles.	A decline in the BDNF.	The BDNF reductions may be due to an enhanced uptake by the brain and its metabolic role in improved cognitive and physical functions.
Noushad et al. [45]	131 (33.14 ± 9.45 years old).	12 weeks (50 min 5 times/week) walking 5 km in a zoo including woodland, mountain viewing, safari tracks, and natural lakes.	The BDNF levels significantly decreased in both walking and sitting groups.	Nature-based walking has a significant positive effect on traumatic stress compared to the sitting group.
Reed et al. [46]	135 participants (61 ± 7 years old) with coronary artery disease.	12 weeks of (a) high-intensity interval training, (b) Nordic walking sessions (60 min each), and (c) moderate-to-vigorous intensity continuous training.	No significant main effects were detected in the BDNF concentrations.	Both interventions were beneficial in enhancing physical and mental health for the patients.
Rezola-Pardo et al. [47]	126 participants (≥70 years old).	12 weeks of walking 5–10 min/day in the first month, 15 min/day in the second, and 20 min/day in the third.	No changes in the BDNF were observed.	Not associated with changes in the physical, cognitive, or dual-task performance metrics.
Caserta et al. [48]	24 participants (48.5 ± 10.6 years old).	12 weeks of walking or quadrato motor training.	Daily quadrato motor training practice increased proNGF, in contrast to simple walking training.	—
Walentukiewicz et al. [49]	94 women (68 ± 5.12 years old).	12 weeks of Nordic walking (45–55 min without warm up/cool down) with vitamin D. (a) Nordic walking (NW), (b) supplemented (SG), (c) control.	The BDNF did not change after the first NW session, but a single session of NW after 12 weeks decreased the BDNF. In both groups, a drop in the BDNF was noted. The change in the BDNF was higher in the SG group (55%) compared to the NW (25%).	Changes in the blood BDNF concentration may not necessarily reflect an increase in the BDNF in the brain after physical activity, potentially signifying a reduced BDNF release by the brain or its higher uptake.
Gmiąt et al. [50]	35 women (68 ± 5.12 years old).	12 weeks of Nordic walking with vitamin D (40 min without warm up and cool down) 3 times/week.	Increased irisin and BDNF concentration.	Improvement of cognitive functions.

**Table 4:** Overview of included long-term studies between 24 and 52 weeks.

Author/s and Publication Year	Sample	Study Design (24 to 52 Weeks)	Results	Discussion Highlights
Chou et al. [51]	58 (69.5 ± 7 years old) hypertensive women.	24-week aerobic walking (30 min of moderate-intensity walking 5 times/week).	The walking group had no significant effect on the BDNF.	Improvement in total recall, delayed recall, and subjective cognitive impairment.
Leckie et al. [52]	90 women (68.82 ± 5.59 years old).	1 year (48 weeks) walking intervention (increased duration by 5 min until reaching 40 min in week 7).	Age moderated the increase in the BDNF levels, specifically those above 71.	The BDNF mediated the effect of exercise on task-switch performance only for individuals over the age of 71. Age and BDNF are associated.
Voss et al. [53]	65 participants (Age_mean_ = 66.4 years old).	1 year (48 weeks) aerobic walking programme (3 times/week; increased duration by 5 min until reaching 40 min in week 7).	No group-level changes in growth factors.	There was an increase in the temporal lobe connectivity between the bilateral parahippocampal and the bilateral middle temporal gyrus, and it was associated with increased BDNF, IGF-1, and VEGF for an aerobic walking group only.
Bergman et al. [54]	80 participants (40–67 years old).	13 months (52 weeks), during which treadmill workstations were installed in offices for light-intensity walking.	Positive associations are found between light-intensity walking and changes in hippocampal volume, but they are not mediated by BDNF changes.	Intensity levels can affect the changes in the BDNF.

**Table 5:** Significance and effect estimates of BDNF changes in response to walking (single bout).

Reference	Age (Years)	Walking Intervention	Duration	BDNF Medium	*p*-Value	Effect Estimate
Hutchinson et al. [35]	18 to 40	Moderate intensity (40–60% heart rate reserve).	30 min	Serum	0.025 (pregnant) and <0.0001 (non-pregnant).	*F* = 35.89.
Michalik et al. [36]	27.6 ± 6.1	6 km/h, (a) with a face mask, (b) without a mask. 7 days apart.	30 min	Serum	Not sig.	—
Silveira-Rodrigues et al. [37]	63 ± 7	4.9 ± 0.9 km/h (90–95% of the maximum walking speed).	40 min	Plasma	Not sig.	+11%; *d* = 0.30.
Goulet et al. [38]	Varies	200 W/m^2^.	3 h	Serum	≤0.01 (32 °C).	+1106 pg/mL (end exercise); +938 pg/mL (end recovery).
Kettinen et al. [39]	69 ± 4	(a) 18-hole golf round, (b) 6 km Nordic walking, and (c) 6 km walking.	3.5 h	Serum	(a) 0.391, (b) 0.968 (0.046 after 40 h), and (c) 0.523.	—
Yasuoka et al. [34]	31 ± 4	18-hole round of golf walk.	5 h	Serum	0.038.	1.2 ± 0.3-fold immediately post exercise.
Wheeler et al. [40]	67 ± 7	SIT, EX + SIT, EX + BR.	8 h	Serum	EX + SIT = 0.03; EX + BR = 0.045.	EX + SIT vs. SIT = (+171 (−449 to +791)); EX + BR vs. SIT = (+139 (−481 to +759)).

**Table 6:** Comparison of changes in BDNF in response to walking (short term, 2 weeks).

Reference	Age (Years)	Walking Intervention	Duration	BDNF Medium	*p*-Value	Effect Estimate	Adaptive Outcomes
Otsuka et al. [41]	>65	Low to moderate intensity walking (5732.1 ± 2829.8 steps/day).	2 weeks	Serum	0.246	—	White matter hyperintensities (faster longer walking).
Yulinda et al. [42]	Age_mean_ = 31	3 km/h speed, while inclination is gradually increased from 2.5% to 22%.	2 weeks	Serum	Inclination: >0.05; Speed: 0.001 (first to last), and 0.159 (first day).	Speed: +111% (first to last).	—

**Table 7:** Comparison of changes in BDNF in response to walking (long term, 8–12 weeks).

Reference	Age (Years)	Walking Intervention	Duration	BDNF Medium	*p*-Value	Effect Estimate	Adaptive Outcomes
Domaszewska et al. [43]	60–75	Twice/week (3.5 to 4.5 km), (a) Nordic walking w/classic poles or (b) Nordic walking w/poles and a resistance shock absorber.	8 weeks	Serum	Nordic walking ≤ 0.05	ES: 0.11 (−1.53 ± 5.04)	Improved cardiopulmonary efficiency.
Rodziewicz-Flis et al. [44]	68.7 ± 6	3 times/day Nordic walking training with BungyPump resistance poles.	12 weeks	Serum	0.02	−16.7% (after 12 weeks)	Improved cognitive functions and physical performance (Nordic walking)
Noushad et al. [45]	33.14 ± 9.45	50 min 5 times/week walking 5 km in nature.	12 weeks	—	<0.01	η^2^ = 0.065	Improved outcomes related to traumatic stress.
Reed et al. [46]	61 ± 7	Nordic walking for 60 min twice weekly.	12 weeks	Plasma	Not sig.	−0.4 ± 7.7	Better functional capacity and quality of life, and reduced depression.
Rezola-Pardo et al. [47]	≥70	Walking 5–10 min/day in the 1st month, 15 min/day in the 2nd, and 20 min/day in the 3rd.	12 weeks	Serum	Not sig.	−682 (11%)	Improved physical and dual-task performance and preserving cognitive function.
Caserta et al. [48]	48.5 ± 10.6	Walking or quadrato motor training.	12 weeks	Saliva	Not sig.	—	—
Walentukiewicz et al. [49]	68 ± 5.12	3 times/week 45–55 min of Nordic walking at 60–70% intensity of the maximal heart rate (HR).	12 weeks	Serum	Not sig.	25% drop	—
Gmiąt et al. [50]	68 ± 5.12	3 times/week Nordic walking (40 min without warm up/cool down) at 60–70% intensity of the maximal HR.	12 weeks	Serum	Likely	After the 1st walk, −6% for beginners, and +31% for advanced individuals	Improved cognitive functions.

**Table 8:** Comparison of changes in BDNF in response to walking (long term, 24–52 weeks).

Reference	Age (Years)	Walking Intervention	Duration	BDNF Medium	*p*-Value	Effect Estimate	Adaptive Outcomes
Chou et al. [51]	69.5 ± 7	30 min walking of moderate intensity 5 times/week.	24 weeks	Plasma	Not sig.	—	Improved recall and subjective cognitive impairment.
Leckie et al. [52]	68.82 ± 5.59	Moderate-intensity walking for 10 min, increasing duration weekly by 5 min until reaching 40 min in week 7.	1 year	Serum	0.036 (with age)	B = 471.95	Improved performance on a task-switching paradigm.
Voss et al. [53]	mean = 66.4	3 times/week. Started walking for 10 min, increasing duration weekly by 5 min until reaching 40 min in week 7.	1 year	Serum	<0.05	τ = 0.25 (with the plasticity change)	Better connectivity between the bilateral parahippocampal and bilateral middle temporal gyrus is associated with an elevated BDNF.
Bergman et al. [54]	40 to 67	Light-intensity walking on treadmills.	13 months	—	Not sig.	—	Changes in hippocampal volume, not mediated by the BDNF.

**Table 9:** Risk of bias scores for other non-randomised studies using the ROBINS-I tool.

Study	ROBINS-I Tool							
	D1	D2	D3	D4	D5	D6	D7	Overall
Otsuka et al. [41]	?	?	+	+	?	+	+	?
Rodziewicz-Flis et al. [44]	?	?	+	+	+	+	+	?
Yasuoka et al. [34]	?	?	+	+	+	+	+	?
Hutchinson et al. [35]	?	?	+	+	+	+	+	?
Gmiąt et al. [50]	?	?	+	+	+	+	+	?

Domains: D1 = bias due to confounding, D2 = bias due to selection of participants. D3 = bias in classification of interventions, D4 = bias due to deviations from intended interventions, D5 = bias due to missing data, D6 = bias in measurement of outcomes, D7 = bias in selection of the reported result. Assessment is as follows: + = low risk of bias, ? = moderate risk of bias, x = serious risk of bias, and ! = critical risk of bias.

**Table 10:** Risk of bias scores for randomised controlled studies using the PEDro scale.

Study	PEDro Scale Items										
	1	2	3	4	5	6	7	8	9	10	Total Score
Kettinen et al. [39]	Y	N	Y	N	N	N	Y	Y	Y	Y	6
Michalik et al. [36]	Y	N	Y	N	N	N	Y	Y	Y	Y	6
Silveira-Rodrigues et al. [37]	N	N	Y	N	N	Y	N	Y	Y	Y	6
Goulet et al. [38]	Y	N	Y	N	N	N	Y	Y	Y	Y	6
Chou et al. [51]	Y	N	Y	N	N	N	N	Y	Y	Y	5
Noushad et al. [45]	Y	Y	Y	N	N	N	Y	Y	Y	Y	7
Reed et al. [46]	Y	Y	Y	N	Y	Y	Y	Y	Y	Y	9
Domaszewska et al. [43]	Y	Y	Y	N	Y	Y	N	Y	Y	Y	8
Rezola-Pardo et al. [47]	Y	N	Y	N	N	Y	N	Y	Y	Y	6
Bergman et al. [54]	Y	N	Y	N	N	N	Y	Y	Y	Y	6
Wheeler et al. [40]	Y	N	Y	N	N	Y	Y	Y	Y	Y	7
Caserta et al. [48]	Y	N	Y	N	N	N	N	Y	Y	Y	5
Yulinda et al. [42]	Y	N	Y	N	N	N	Y	Y	Y	Y	6
Walentukiewicz et al. [49]	Y	N	Y	N	N	N	Y	Y	Y	Y	6
Leckie et al. [52]	Y	N	Y	N	N	N	Y	Y	Y	Y	6
Voss et al. [53]	Y	N	Y	N	N	N	Y	Y	Y	Y	6

PEDro scale items: 1 = random allocation, 2 = concealed allocation, 3 = groups similar at baseline, 4 = participant blinding, 5 = therapist blinding, 6 = assessment blinding, 7 = <15% dropout rate, 8 = intention-to-treat analysis, 9 = between-group differences reported, 10 = point estimate and variability reported. Y = yes. N = no. According to the scale, scores 0–3 are ’poor’, 4–5 are ’fair’, 6–8 are ’good’, and 9–10 are ’excellent’.

## References

[1] Khalil M.H. (2025). Walking and Hippocampal Formation Volume Changes: A Systematic Review. Brain Sci..

[2] Khalil M.H. (2024). Neurosustainability. Front. Hum. Neurosci..

[3] Khalil M.H. (2024). Environmental Affordance for Physical Activity, Neurosustainability, and Brain Health: Quantifying the Built Environment’s Ability to Sustain BDNF Release by Reaching Metabolic Equivalents (METs). Brain Sci..

[4] Khalil M.H. (2024). The BDNF-interactive model for sustainable hippocampal neurogenesis in humans: Synergistic effects of environmentally-mediated physical activity, cognitive stimulation, and mindfulness. Int. J. Mol. Sci..

[5] Håkansson K., Ledreux A., Daffner K., Terjestam Y., Bergman P., Carlsson R., Kivipelto M., Winblad B., Granholm A.C., Mohammed A.K.H. (2016). BDNF responses in healthy older persons to 35 minutes of physical exercise, cognitive training, and mindfulness: Associations with working memory function. J. Alzheimer’s Dis..

[6] Park S.A., Lee A.Y., Park H.G., Lee W.L. (2019). Benefits of gardening activities for cognitive function according to measurement of brain nerve growth factor levels. Int. J. Environ. Res. Public Health.

[7] Dinoff A., Herrmann N., Swardfager W., Lanctot K.L. (2017). The effect of acute exercise on blood concentrations of brain-derived neurotrophic factor in healthy adults: A meta-analysis. Eur. J. Neurosci..

[8] Szuhany K.L., Bugatti M., Otto M.W. (2015). A meta-analytic review of the effects of exercise on brain-derived neurotrophic factor. J. Psychiatr. Res..

[9] Wang Y.H., Zhou H.H., Luo Q., Cui S. (2022). The effect of physical exercise on circulating brain-derived neurotrophic factor in healthy subjects: A meta-analysis of randomized controlled trials. Brain Behav..

[10] Antunes B.M., Rossi F.E., Teixeira A.M., Lira F.S. (2020). Short-time high-intensity exercise increases peripheral BDNF in a physical fitness-dependent way in healthy men. Eur. J. Sport Sci..

[11] Walsh E.I., Smith L., Northey J., Rattray B., Cherbuin N. (2020). Towards an understanding of the physical activity-BDNF-cognition triumvirate: A review of associations and dosage. Ageing Res. Rev..

[12] Rodríguez-Gutiérrez E., Torres-Costoso A., Saz-Lara A., Bizzozero-Peroni B., Guzmán-Pavón M.J., Sánchez-López M., Martínez-Vizcaíno V. (2024). Effectiveness of high-intensity interval training on peripheral brain-derived neurotrophic factor in adults: A systematic review and network meta-analysis. Scand. J. Med. Sci. Sports.

[13] Zhou B., Wang Z., Zhu L., Huang G., Li B., Chen C., Huang J., Ma F., Liu T.C. (2022). Effects of different physical activities on brain-derived neurotrophic factor: A systematic review and bayesian network meta-analysis. Front. Aging Neurosci..

[14] Numakawa T., Odaka H., Adachi N. (2018). Actions of brain-derived neurotrophin factor in the neurogenesis and neuronal function, and its involvement in the pathophysiology of brain diseases. Int. J. Mol. Sci..

[15] Cattaneo A., Cattane N., Begni V., Pariante C.M., Riva M.A. (2016). The human BDNF gene: Peripheral gene expression and protein levels as biomarkers for psychiatric disorders. Transl. Psychiatry.

[16] Pruunsild P., Kazantseva A., Aid T., Palm K., Timmusk T. (2007). Dissecting the human BDNF locus: Bidirectional transcription, complex splicing, and multiple promoters. Genomics.

[17] Je H.S., Yang F., Ji Y., Potluri S., Fu X.Q., Luo Z.G., Nagappan G., Chan J.P., Hempstead B., Son Y.J. (2013). ProBDNF and mature BDNF as punishment and reward signals for synapse elimination at mouse neuromuscular junctions. J. Neurosci..

[18] Lu B. (2003). Pro-region of neurotrophins: Role in synaptic modulation. Neuron.

[19] Chao M.V. (2003). Neurotrophins and their receptors: A convergence point for many signalling pathways. Nat. Rev. Neurosci..

[20] Hua Z., Gu X., Dong Y., Tan F., Liu Z., Thiele C.J., Li Z. (2016). PI3K and MAPK pathways mediate the BDNF/TrkB-increased metastasis in neuroblastoma. Tumor Biol..

[21] Li Y., Wei C., Wang W., Li Q., Wang Z.C. (2023). Tropomyosin receptor kinase B (TrkB) signalling: Targeted therapy in neurogenic tumours. J. Pathol. Clin. Res..

[22] Sun Y., Liu W.Z., Liu T., Feng X., Yang N., Zhou H.F. (2015). Signaling pathway of MAPK/ERK in cell proliferation, differentiation, migration, senescence and apoptosis. J. Recept. Signal Transduct..

[23] Yu J.S., Cui W. (2016). Proliferation, survival and metabolism: The role of PI3K/AKT/mTOR signalling in pluripotency and cell fate determination. Development.

[24] Levy M.J., Boulle F., Steinbusch H.W., van den Hove D.L., Kenis G., Lanfumey L. (2018). Neurotrophic factors and neuroplasticity pathways in the pathophysiology and treatment of depression. Psychopharmacology.

[25] Moher D. (2009). Preferred reporting items for systematic reviews and meta-analyses: The PRISMA statement. Ann. Intern. Med..

[26] Schardt C., Adams M.B., Owens T., Keitz S., Fontelo P. (2007). Utilization of the PICO framework to improve searching PubMed for clinical questions. BMC Med. Inform. Decis. Mak..

[27] Farrugia P., Petrisor B.A., Farrokhyar F., Bhandari M. (2010). Practical tips for surgical research: Research questions, hypotheses and objectives. Can. J. Surgery. J. Can. Chir..

[28] De Morton N.A. (2009). The PEDro scale is a valid measure of the methodological quality of clinical trials: A demographic study. Aust. J. Physiother..

[29] Sterne J.A., Hernán M.A., Reeves B.C., Savović J., Berkman N.D., Viswanathan M., Henry D., Altman D.G., Ansari M.T., Boutron I. (2016). ROBINS-I: A tool for assessing risk of bias in non-randomised studies of interventions. BMJ.

[30] Bos I., De Boever P., Vanparijs J., Pattyn N., Panis L.I., Meeusen R. (2013). Subclinical effects of aerobic training in urban environment. Med. Sci. Sports Exerc..

[31] Máderová D., Krumpolec P., Slobodová L., Schön M., Tirpáková V., Kovaničová Z., Klepochová R., Vajda M., Šutovský S., Cvečka J. (2019). Acute and regular exercise distinctly modulate serum, plasma and skeletal muscle BDNF in the elderly. Neuropeptides.

[32] Yoshino K., Umeno A., Shichiri M., Watanabe H., Ishida N., Kojima M., Iwaki S., Hagihara Y., Nakamura M., Yoshida Y. (2015). Biomarkers for the evaluation of immunological properties during the shikoku walking pilgrimage. J. Biol. Regul. Homeost. Agents.

[33] Schmidt-Kassow M., Zink N., Mock J., Thiel C., Vogt L., Abel C., Kaiser J. (2014). Treadmill walking during vocabulary encoding improves verbal long-term memory. Behav. Brain Funct..

[34] Yasuoka Y., Nakamura T., Umemoto Y., Kinoshita T., Hoekstra S.P., Hoshiai K., Ohko H., Abo M., Tajima F. (2022). An 18-hole round of golf acutely elevates serum Interleukin-6 and brain-derived neurotrophic factor concentration—A pilot study. J. Phys. Fit. Sports Med..

[35] Hutchinson K.A., Mohammad S., Garneau L., McInnis K., Aguer C., Adamo K.B. (2019). Examination of the myokine response in pregnant and non-pregnant women following an acute bout of moderate-intensity walking. Front. Physiol..

[36] Michalik K., Smolarek M., Borkowski J., Tchorowski M., Korczuk N., Gorczyca P., Wojtarowicz N., Zatoń M. (2023). Changes in Reaction Time, Balance and Neuroplasticity after Exercise with a Face Mask in Male Adults with Mild COVID-19 Symptoms. Healthcare.

[37] Silveira-Rodrigues J.G., Campos B.T., de Lima A.T., Ogando P.H., Gomes C.B., Gomes P.F., Aleixo I.M., Soares D.D. (2023). Acute bouts of aerobic and resistance exercise similarly alter inhibitory control and response time while inversely modifying plasma BDNF concentrations in middle-aged and older adults with type 2 diabetes. Exp. Brain Res..

[38] Goulet N., McCormick J.J., King K.E., Notley S.R., Goldfield G.S., Fujii N., Amano T., Kenny G.P. (2023). Elevations in serum brain-derived neurotrophic factor following occupational heat stress are not influenced by age or common chronic disease. Temperature.

[39] Kettinen J., Tikkanen H., Hiltunen M., Murray A., Horn N., Taylor W.R., Venojärvi M. (2023). Cognitive and biomarker responses in healthy older adults to a 18-hole golf round and different walking types: A randomised cross-over study. BMJ Open Sport Exerc. Med..

[40] Wheeler M.J., Green D.J., Ellis K.A., Cerin E., Heinonen I., Naylor L.H., Larsen R., Wennberg P., Boraxbekk C.J., Lewis J. (2020). Distinct effects of acute exercise and breaks in sitting on working memory and executive function in older adults: A three-arm, randomised cross-over trial to evaluate the effects of exercise with and without breaks in sitting on cognition. Br. J. Sports Med..

[41] Otsuka S., Kikuchi K., Takeshita Y., Takada S., Tani A., Sakakima H., Maruyama I., Makizako H. (2024). Relationship between physical activity and cerebral white matter hyperintensity volumes in older adults with depressive symptoms and mild memory impairment: A cross-sectional study. Front. Aging Neurosci..

[42] Yulinda S.T., Tinduh D., Wardhani L., Laswati H., Wibisono S., Soenarnatalina M. (2019). Brain Derived Neurotropic Factors in Speed vs. Inclined Treadmill in Young Adult Healthy Male With Occult Balance Disorder. Front. Integr. Neurosci..

[43] Domaszewska K., Koper M., Wochna K., Czerniak U., Marciniak K., Wilski M., Bukowska D. (2020). The effects of Nordic walking with poles with an integrated resistance shock absorber on cognitive abilities and cardiopulmonary efficiency in postmenopausal women. Front. Aging Neurosci..

[44] Rodziewicz-Flis E., Juhas U., Kortas J.A., Jaworska J., Bidzan-Bluma I., Babińska A., Micielska K., Żychowska M., Lombardi G., Antosiewicz J. (2023). Nordic Walking training in BungyPump form improves cognitive functions and physical performance and induces changes in amino acids and kynurenine profiles in older adults. Front. Endocrinol..

[45] Noushad S., Ansari B., Ahmed S. (2022). Effect of nature-based physical activity on post-traumatic growth among healthcare providers with post-traumatic stress. Stress Health.

[46] Reed J.L., Terada T., Cotie L.M., Tulloch H.E., Leenen F.H., Mistura M., Hans H., Wang H.W., Vidal-Almela S., Reid R.D. (2022). The effects of high-intensity interval training, Nordic walking and moderate-to-vigorous intensity continuous training on functional capacity, depression and quality of life in patients with coronary artery disease enrolled in cardiac rehabilitation: A randomized controlled trial (CRX study). Prog. Cardiovasc. Dis..

[47] Rezola-Pardo C., Hervás G., Arrieta H., Hernández-de Diego A., Ruiz-Litago F., Gil S.M., Rodriguez-Larrad A., Irazusta J. (2020). Physical exercise interventions have no effect on serum BDNF concentration in older adults living in long-term nursing homes. Exp. Gerontol..

[48] Caserta M., Ben-Soussan T.D., Vetriani V., Venditti S., Verdone L. (2019). Influence of Quadrato Motor Training on salivary proNGF and proBDNF. Front. Neurosci..

[49] Walentukiewicz A., Lysak-Radomska A., Jaworska J., Prusik K., Prusik K., Kortas J.A., Lipiński M., Babinska A., Antosiewicz J., Ziemann E. (2018). Vitamin D supplementation and Nordic walking training decreases serum homocysteine and ferritin in elderly women. Int. J. Environ. Res. Public Health.

[50] Gmiąt A., Jaworska J., Micielska K., Kortas J., Prusik K., Lipowski M., Radulska A., Szupryczyńska N., Antosiewicz J., Ziemann E. (2018). Improvement of cognitive functions in response to a regular Nordic walking training in elderly women—A change dependent on the training experience. Exp. Gerontol..

[51] Chou C.C., Chien L.Y., Lin M.F., Wang C.J., Liu P.Y. (2022). Effects of Aerobic Walking on Memory, Subjective Cognitive Complaints, and Brain-Derived Neurotrophic Factor Among Older Hypertensive Women. Biol. Res. Nurs..

[52] Leckie R.L., Oberlin L.E., Voss M.W., Prakash R.S., Szabo-Reed A., Chaddock-Heyman L., Phillips S.M., Gothe N.P., Mailey E. (2014). BDNF mediates improvements in executive function following a 1-year exercise intervention. Front. Hum. Neurosci..

[53] Voss M.W., Erickson K.I., Prakash R.S., Chaddock L., Kim J.S., Alves H., Szabo A., Phillips S.M., Wójcicki T.R., Mailey E.L. (2013). Neurobiological markers of exercise-related brain plasticity in older adults. Brain Behav. Immun..

[54] Bergman F., Matsson-Frost T., Jonasson L., Chorell E., Sörlin A., Wennberg P., Öhberg F., Ryberg M., Levine J.A., Olsson T. (2020). Walking time is associated with hippocampal volume in overweight and obese office workers. Front. Hum. Neurosci..

[55] Molendijk M.L., Haffmans J.P., Bus B.A., Spinhoven P., Penninx B.W., Prickaerts J., Voshaar R.C.O., Elzinga B.M. (2012). Serum BDNF concentrations show strong seasonal variation and correlations with the amount of ambient sunlight. PLoS ONE.

[56] Kojima D., Nakamura T., Banno M., Umemoto Y., Kinoshita T., Ishida Y., Tajima F. (2018). Head-out immersion in hot water increases serum BDNF in healthy males. Int. J. Hyperth..

[57] Koyama Y., Mukuda T., Hamasaki S., Nakane H., Kaidoh T. (2018). Short-term heat exposure promotes hippocampal neurogenesis via activation of angiotensin II type 1 receptor in adult rats. Neuroscience.

[58] Sudimac S., Kühn S. (2022). A one-hour walk in nature reduces amygdala activity in women, but not in men. Front. Psychol..

[59] Bath K.G., Schilit A., Lee F.S. (2013). Stress effects on BDNF expression: Effects of age, sex, and form of stress. Neuroscience.

[60] Ploughman M., Austin M.W., Glynn L., Corbett D. (2015). The effects of poststroke aerobic exercise on neuroplasticity: A systematic review of animal and clinical studies. Transl. Stroke Res..

[61] de Sousa Fernandes M.S., Ordônio T.F., Santos G.C.J., Santos L.E.R., Calazans C.T., Gomes D.A., Santos T.M. (2020). Effects of physical exercise on neuroplasticity and brain function: A systematic review in human and animal studies. Neural Plast..

[62] Johansson H., Hagströmer M., Grooten W.J., Franzén E. (2020). Exercise-induced neuroplasticity in Parkinson’s disease: A metasynthesis of the literature. Neural Plast..

[63] Rotondo R., Proietti S., Perluigi M., Padua E., Stocchi F., Fini M., Stocchi V., Volpe D., De Pandis M.F. (2023). Physical activity and neurotrophic factors as potential drivers of neuroplasticity in Parkinson’s Disease: A systematic review and meta-analysis. Ageing Res. Rev..

[64] Penna L.G., Pinheiro J.P., Ramalho S.H.R., Ribeiro C.F. (2021). Effects of aerobic physical exercise on neuroplasticity after stroke: Systematic review. Arq. Neuro-Psiquiatr..

[65] Sandroff B.M., Jones C.D., Baird J.F., Motl R.W. (2020). Systematic review on exercise training as a neuroplasticity-inducing behavior in multiple sclerosis. Neurorehabilit. Neural Repair.

[66] Cámara-Calmaestra R., Martínez-Amat A., Aibar-Almazán A., Hita-Contreras F., de Miguel Hernando N., Achalandabaso-Ochoa A. (2022). Effectiveness of physical exercise on Alzheimer’s disease. A systematic review. J. Prev. Alzheimer’s Dis..

[67] Guo L., Yang X., Zhang Y., Xu X., Li Y. (2023). Effect of exercise on cognitive function and synaptic plasticity in Alzheimer’s disease models: A systematic review and meta-analysis. Front. Aging Neurosci..

[68] Lin T.W., Tsai S.F., Kuo Y.M. (2018). Physical exercise enhances neuroplasticity and delays Alzheimer’s disease. Brain Plast..

[69] Cardoso S.V., Fernandes S.R., Tomás M. (2024). Therapeutic importance of exercise in neuroplasticity in adults with neurological pathology: Systematic review. Int. J. Exerc. Sci..

[70] Knaepen K., Goekint M., Heyman E.M., Meeusen R. (2010). Neuroplasticity—Exercise-induced response of peripheral brain-derived neurotrophic factor: A systematic review of experimental studies in human subjects. Sports Med..

[71] Pan W., Banks W.A., Fasold M.B., Bluth J., Kastin A.J. (1998). Transport of brain-derived neurotrophic factor across the blood–brain barrier. Neuropharmacology.

[72] Poduslo J.F., Curran G.L. (1996). Permeability at the blood-brain and blood-nerve barriers of the neurotrophic factors: NGF, CNTF, NT-3, BDNF. Mol. Brain Res..

[73] Varma V.R., Tang X., Carlson M.C. (2016). Hippocampal sub-regional shape and physical activity in older adults. Hippocampus.

[74] Zabetian-Targhi F., Srikanth V.K., Beare R., Breslin M., Moran C., Wang W., Wu F., Smith K.J., Callisaya M.L. (2021). The association between physical activity intensity, cognition, and brain structure in people with type 2 diabetes. J. Gerontol. Ser. A.

[75] Fotuhi M., Do D., Jack C. (2012). Modifiable factors that alter the size of the hippocampus with ageing. Nat. Rev. Neurol..

[76] Brunoni A.R., Lopes M., Fregni F. (2008). A systematic review and meta-analysis of clinical studies on major depression and BDNF levels: Implications for the role of neuroplasticity in depression. Int. J. Neuropsychopharmacol..

[77] Yang T., Nie Z., Shu H., Kuang Y., Chen X., Cheng J., Yu S., Liu H. (2020). The role of BDNF on neural plasticity in depression. Front. Cell. Neurosci..

[78] Khalil M.H., Steemers K. (2024). Housing Environmental Enrichment, Lifestyles, and Public Health Indicators of Neurogenesis in Humans: A Pilot Study. Int. J. Environ. Res. Public Health.

[79] Khalil M.H. (2025). Borderline in a linear city: Urban living brings borderline personality disorder to crisis through neuroplasticity—an urgent call to action. Front. Psychiatry.

